# Do you know this syndrome? Type 2 benign symmetric lipomatosis (Launois-Bensaude)*

**DOI:** 10.1590/abd1806-4841.20164744

**Published:** 2016

**Authors:** Ana Cláudia Cavalcante Esposito, Tania Munhoz, Luciana Patrícia Fernandes Abbade, Hélio Amante Miot

**Affiliations:** 1 Universidade Estadual Paulista "Júlio de Mesquita Filho" (Unesp) – Botucatu (SP), Brazil

**Keywords:** lipoma, lipomatosis, multiple symmetric lipomatosis

## Abstract

A 57-year-old female showed bulky, loose tumors, which progressively spread to her arms, anterior chest, and back. She reported dysphagia and dyspnea after mild exertion. She denied alcohol consumption. CT scan of her chest showed no internal lesions. Benign symmetric lipomatosis is a rare syndrome, clinically described as multiple nonencapsulated lipomas of various sizes and symmetrical distribution. This syndrome has three known phenotypes; in type 2 (Launois-Bensaude syndrome), lesions occur primarily on the shoulders, upper arms, and chest, and is unrelated to alcoholism. It causes aesthetic deformities and might block the upper airways. Mediastinal invasion might occur as well.

## CASE REPORT

**Figure 1:**
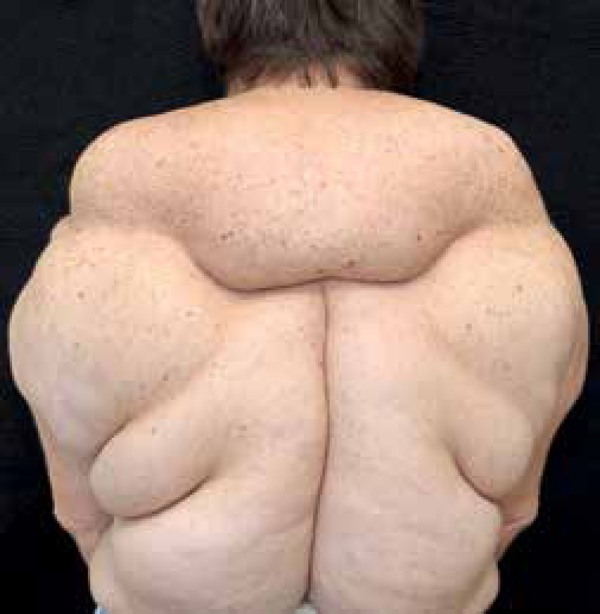
Soft, symmetrical, bulky tumors on the back

**Figure 2:**
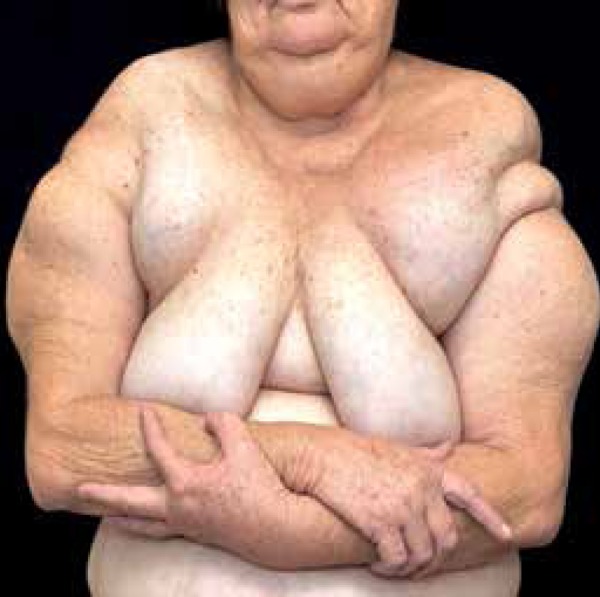
Tumors on the chest and shoulders, giving patient a "pseudo-athletic" shape

**Figure 3:**
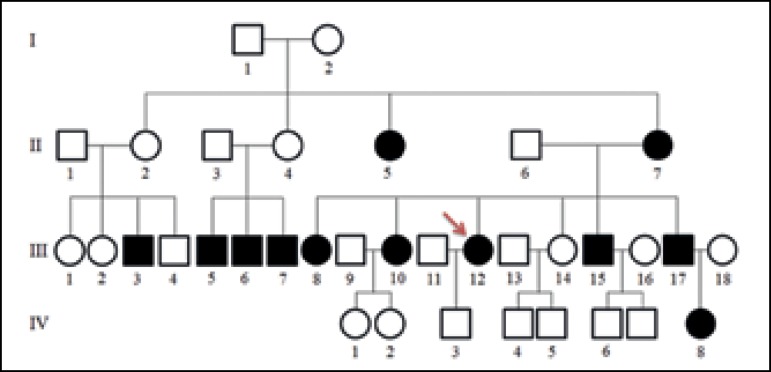
Heredogram. The arrow indicates the reported case (III-12)

A 57-year-old Caucasian female patientpresented with slow growing nodes that had been present for the last 24 years, at first on her arms, and currently diffusely distributed on her body (Figures 1 and 2). She complained of progressive dysphagia and dyspnea after small exertion, and reported well-controlled hypertension. She denied alcohol abuse. She underwent liposuction for eight similar lesions, with subsequent local recurrence. First and second degree relatives were reported to have similar lesions with different degrees of involvement (Figure 3). Dermatological examination showed symmetrical, painless tumors, of stiff, smooth, and soft texture, located on her arms, anterior thorax, trapezius, and along her back. Lab tests showed hypertriglyceridemia (247 IU/ml). CT scan (with contrast) of the chest showed diffuse enhancement of soft tissues (fat density) without internal lesions.

## DISCUSSION

Benign symmetric lipomatosis (BSL), or multiple symmetric lipomatosis, is a rare syndrome (1:25.000) that affects 30-60 year-old people, specially men (15-30:1) and alcoholics.^1^ Familial cases with autosomal dominant inheritance have been reported.^2^ BSL pathogenesis is still unclear, but involves proliferation of functionally defective brown adipocytes, whether by mitochondrial disorders or by AMPc dysfunction.^3-4^

It is characterized by multiple nonencapsulated symmetrical lipomas with various diameters (1-20cm). The patient's face, forearms, and distal third of the leg are usually intact. Lesions grow in a slow, progressively manner, into unsightly deformities. Upper airway obstruction and mediastinal invasion might occur as well.^5^ Patients often show metabolic syndrome (diabetes mellitus, hyperlipidemia, and hyperuricemia), as well as peripheral sensory and motor neuropathy.^6^

BSL has three phenotypes, based on the anatomical location of the lesions. Type 1 (Madelung disease): Lipomatosis is located primarily in the cervical region, in kyphosis or "horse collar" manner, and is associated with alcoholism. Type 2 (Launois-Bensaude syndrome – LBS): It is unrelated to alcoholism; lesions occur primarily on shoulders, upper arms, and chest (sometimes in the abdomen and the back), giving the patient an "pseudo-athtletic" shape. Type 3 (gynecoid): Lipomatosis occurs primarily on the pelvic waist.^7^

LSB diagnosis is clinical; imaging exams (primarily CT scan) are important to assess the extent of lipomatosis, to rule out differential diagnosis, and to support pre-operative exams.^8^ Aspiration of lesions shows only fat cells. Lipoma, angiolipoma, liposarcoma, multiple family lipomatosis, and Dercum's disease are differential diagnoses of LSB.^9^

Surgery (resection or liposuction) is the most effective treatment, due to the aesthetic deformities and compressive symptoms.^8^ No treatments may prevent the formation of new lesions. Decreased progression with salbutamol (12 mg/day), as a result of lipolysis by adrenergic stimulation, has been reported.^10^ The outcome of cases is not affected by dietary changes.

In this case, the diagnosis of type 2 LSB (Launois-Bensaude syndrome) was raised by its typical distribution (trunk and proximal region of the upper limbs) and no personal history of alcohol abuse. Genetic exam infers a pattern of autosomal dominant inheritance with incomplete penetrance.
